# An immunoproteomic approach reveals a different pattern of non-infected erythrocyte membrane protein recognition by antibodies from non-anemic and anemic patients with patent *Plasmodium vivax* infection

**DOI:** 10.1186/1475-2875-13-S1-P17

**Published:** 2014-09-22

**Authors:** Luiza Mourao, Maira Pucci, Zelia Almeida, Rosiane Pereira, Cor Jesus Fontes, Marcus Lacerda, Marcelo Bemquerer, Erika Braga

**Affiliations:** 1 Universidade Federal de Minas Gerais, Belo Horizonte, Minas Gerais, Brazil; 2 Centro de Pesquisas Rene Rachou, Belo Horizonte, Minas Gerais, Brazil; 3 Universidade Federal de Mato Grosso, Cuiaba, Mato Grosso, Brazil; 4 Fundação de Medicina Tropical Dr. Heitor Vieira Dourado, Manaus, Amazonas, Brazil; 5 Embrapa Recursos Genéticos e Biotecnologia, Brasilia, Distrito Federal, Brazil

## 

Anemia is a typical manifestation of *Plasmodium vivax* infection and one of the leading causes of morbidity and mortality, particularly in pregnant women and children less than five years old. Despite the magnitude of the problem, there remain major unsolved questions about its etiology. In this context, we have addressed the following question: Are antibodies directed to erythrocyte membrane proteins involved in the accelerated clearance of red blood cells (RBCs) by phagocytes leading to anemia during *P. vivax* infection? We performed immunoproteomic assays trying to characterize proteins that are targets of antibodies. Sera from four groups of subjects were tested against a non-infected O+ erythrocyte membrane extract and are included in our study: *P. vivax* anemic individuals (PvAN); *P. vivax* non-anemic individuals (PvNA); non-infected individuals with anemia by other etiologies (NIAN); and non-infected and non-anemic individuals (NINA). Immunoblots revealed a differential pattern of RBC protein recognition related to the four studied groups. The breadth and the magnitude of antibody responses against RBC membrane antigens were higher in NAPv and ANPv, corroborating our previous results obtained by ELISA and 1-D immunoblot. Now, these proteins are being identified by peptide mass fingerprint. Next, we will confirm their identity by MALDI TOF/MS-MS, validate and categorize them in relation to location, family and function. Highly immunogenic and differentially recognized proteins will represent potential candidates for diagnostic or prognostic biomarkers of anemia in *P. vivax* infections. Moreover, data generated here will contribute to the better understanding of mechanisms enrolled in *vivax* anemia pathogenesis.

